# Diet Quality of Independently Living Older Adults in Massachusetts (2021-2025)

**DOI:** 10.1093/geroni/igaf122.4100

**Published:** 2025-12-31

**Authors:** Qun Le, Lingming Chen, Danielle LoPilato, Stephanie Chavarria, Jie Cheng, Meng Zhang, Sabrina E Noel, Wenjun Li

**Affiliations:** University of Massachusetts Lowell, Lowell, Massachusetts, United States; University of Massachusetts Lowell, Lowell, Massachusetts, United States; University of Massachusetts Lowell, Lowell, Massachusetts, United States; University of Massachusetts Lowell, Lowell, Massachusetts, United States; University of Massachusetts Lowell, Lowell, Massachusetts, United States; University of Massachusetts Lowell, Lowell, Massachusetts, United States; University of Massachusetts Lowell, Lowell, Massachusetts, United States; University of Massachusetts Lowell, Lowell, Massachusetts, United States

## Abstract

A healthy diet is key to healthy aging. Access to nutritious food is one of the major social determinants of health in Healthy People 2030. In this analysis, associations between dietary quality and factors such as age, race, and other personal and environmental characteristics were quantified. The Healthy Aging and Neighborhood Study (2021-2025) collected sociodemographic and dietary data from 614 people aged 65 y and older living in communities in Central and Northeastern Massachusetts. Dietary quality was evaluated by the Dietary Screening Tool (range 0-100) with higher scores indicating higher diet quality. Generalized linear regression models were used and regression coefficients (B(SE)) were presented. The cohort was on average aged 74.6(±6.2) y, 57.7% female, 56.0% Non-Hispanic White, 32.9% Asian, and 8.6% Hispanic. Without covariate adjustment, higher diet quality was significantly associated with older age (B(SE) =0.2(0.1)), Asian race (9.7(1.0)), having any difficulty in IADL (2.8(1.0)), being married (2.4(1.0)), and living in suburban areas (5.2(1.5)), and urban areas (5.0(1.3)). Similarly, lower diet quality was related to higher educational attainment (-0.4(0.2)), household income over $50,000/year (-4.8(1.1)), and better self-rated general health (-1.4(0.4)). However, after covariate adjustments, higher diet quality was associated only with female gender (2.3(1.0)) and Asian race (13.0(1.8)). The changes in directions and strengths of the associations indicate the complicated relationships between sociodemographic factors and diet quality in older age. Future research should explore the mechanisms of sociodemographic differences in dietary quality.

